# Serum β2-Microglobulin Is Closely Associated With the Recurrence Risk and 3-Month Outcome of Acute Ischemic Stroke

**DOI:** 10.3389/fneur.2019.01334

**Published:** 2020-01-09

**Authors:** Fu-yong Hu, Juncang Wu, Qiqiang Tang, Ji Zhang, Zhengxu Chen, Xiaoqiang Wang, Qiuwan Liu, Juan Wang, Wei Ge, Sen Qun

**Affiliations:** ^1^Stroke Center & Department of Neurology, The First Affiliated Hospital of USTC, Division of Life Sciences and Medicine, University of Science and Technology of China, Hefei, China; ^2^School of Public Health, Bengbu Medical College, Bengbu, China; ^3^Department of Neurology, The No. 2 People's Hospital of Hefei, Hefei, China; ^4^Department of Neurology, The Affiliated Hospital of Xuzhou Medical University, Xuzhou, China

**Keywords:** β2-microglobulin, acute ischemic stroke, 3-month outcome, risk of recurrence, inflammation

## Abstract

**Background and Purpose:** Inflammation plays a significant role in the pathogenesis of acute ischemic stroke (AIS). The role of β2-microglobulin (β2M) as a potential initiator of the inflammatory response in AIS is unclear. The purpose of this study was to analyze the relationship of serum β2M with the recurrence risk and 3-month outcome of AIS.

**Methods:** A total of 205 patients with AIS were recruited, and their clinical and biochemical characteristics were collected. All patients were followed up for 3 months after stroke onset, and the occurrence of death or major disability at 3 months after onset was the outcome of interest in this study. We evaluated the association of serum β2M levels with the National Institute of Health Stroke Scale (NIHSS) scores, modified Rankin Scale (mRS) scores, and Essen Stroke Risk Score (ESRS) values in patients with AIS. Then, we used receiver operating curve analysis to calculate the optimal cutoff value for discriminating outcomes in patients with AIS and a binary logistic regression model to evaluate the risk factors for a poor outcome after AIS.

**Results:** Our results showed that serum β2M levels were significantly and positively correlated with ESRS values (*r* = 0.176, *P* < 0.001) and mRS scores (*r* = 0.402, *P* < 0.001), but the levels of β2M were not correlated with NIHSS scores (*r* = 0.080, *P* = 0.255) or with infarct volume (*r* = 0.013, *P* = 0.859). In a further study, we found that 121 patients (59.02%) had poor outcomes. The optimal β2M cutoff to predict the 3-month outcome of AIS in this study was 1.865 mg/l, and β2M was independently associated with a poor outcome at 3 months (*OR* = 3.325, 95% confidence interval: 1.089~10.148).

**Conclusions:** In conclusion, we inferred that serum β2M was positively associated with the recurrence risk and 3-month outcome of AIS, but it did not appear to be directly related to the severity of AIS or the size of the infarct at admission.

## Introduction

Stroke is a global critical public health issue (1). Increasing evidence suggests that one of the major processes worsening the clinical outcome of acute ischemic stroke (AIS) is inflammation (2). Due to the narrow therapeutic time window for the use of tissue plasminogen activator (tPA) in treating AIS, there is still a lack of effective treatment measures (3). In order to shed light on potential therapeutic targets that could improve patient outcomes, a greater understanding of the pathophysiological processes that occur during and following AIS is required. Therefore, our research is focused on the mechanism of inflammation during the AIS period.

β2-microglobulin (β2M) is a small protein (11,800 Da) that is present in nearly all nucleated cells. β2M is a β-chain (light chain) of human lymphocyte antigens (HLAs) on the cell surface and is structurally similar to a region of immunoglobulin (4). As a result of being shed from cell surfaces and being released intracellularly, β2M appears in most biological fluids, including serum, urine, and synovial fluid (5). β2M is a biomarker of kidney disease and function, as the plasma level of β2M is increased when the glomerular filtration rate (GFR) is decreased (6). In addition, β2M is a critical component of the major histocompatibility complex class I (MHC I) heterodimer, presenting intracellular antigens to cytotoxic T cells (7). In addition to being a marker of kidney function, β2M has other functions in inflammatory diseases, such as being a potential initiator of inflammatory responses (8). Increased plasma levels of β2M also occur in a variety of autoimmune, neoplastic, infectious, and renal diseases (9). Recent evidence indicated that serum β2M was positively associated with an increased risk of incident ischemic stroke events among women (10). However, the role of β2M in AIS is unclear, and there are few reports on the relationship between β2M and the recurrence or prognosis of ischemic stroke. In this cohort study, we examined the associations between serum β2M and the risk of recurrence and the 3-month outcome of patients with AIS.

## Methods

### Ethical Statements

This study was approved by the Research Ethics Committee of the Hospital of Hefei Affiliated with Anhui Medical University. All participants gave their informed consent prior to their inclusion in this study. Consent was obtained directly from all participants. If participants were considered incapable of giving informed consent themselves, then consent was obtained from their guardians. All participants or their guardians were informed of the purpose of the study and signed informed consent. The written informed consent procedure was approved by the ethics committee.

### Subjects

We collected the clinical data of all inpatients with AIS in the Hospital of Hefei Affiliated with Anhui Medical University from October 2016 to September 2017. The diagnosis of AIS was determined by medical history, symptoms and signs, and diffusion-weighted magnetic resonance imaging (DWI). All patients received anti-platelet medications and statin therapy.

AIS patients who were admitted within 3 days after the onset of stroke symptoms were selected. The exclusion criteria for AIS subjects were as follows: (1) a history of stroke within 6 months and/or pre-stroke disability with a modified Rankin Scale (mRS) score > 0; the presence of (2) severe brain diseases or (3) serious systemic diseases, such as acute or chronic renal dysfunction and endocrine diseases (other than diabetes mellitus); (4) the use of immunosuppressant drugs (steroids); and the presence of (5) cancer, (6) trauma, (7) infectious diseases, (8) hematological disorders, or (9) brainstem infarct, which is known to have a direct effect on mortality [however, malignant middle cerebral artery (MCA) infarction patients were included in the research cohort].

### Laboratory Assays and Clinical Information

The blood samples for laboratory tests were collected on the morning of the second day after admission (between 6:00 and 7:00 a.m.) following an overnight fast. Serum β2M was measured by the particle-enhanced turbidimetric immunoassay method. The intra-assay coefficient of variation ranged from 2.4 to 3.8%, and the inter-assay coefficient of variation ranged from 1.7 to 2.2%. C-reactive protein (CRP) was measured by the immune transmission turbidity method. Other biochemical parameters, such as creatinine (Cr), urea, and triglyceride (TG), were measured by enzymatic methods. All serum biochemical parameters were assayed using an automatic biochemical analyzer (HITACHI Automatic Analyzer 7600-020, Japan).

At baseline, we collected the demographic characteristics of age and gender, as well as information on the presence of cerebral vascular risk factors such as hypertension and diabetes, from all participants. Hypertension was identified by the previous use of antihypertensive medication, systolic blood pressure (SBP) ≥ 140 mmHg, or diastolic blood pressure (DBP) ≥ 90 mmHg. Diabetes was identified by the previous use of anti-diabetic medication, fasting blood glucose ≥ 7.0 mmol/l, or 2-h postprandial blood glucose ≥ 11.1 mmol/l.

### Evaluation of 3-Month Outcomes

The follow-up end point was 3 months after stroke onset, and outcomes were evaluated using modified Rankin Scale (mRS) scores (where mRS ≥ 3, up to and including death, was defined as a poor outcome) (11). Data were obtained from hospital records or by telephone interviews with patients, their relatives, or their family physicians.

### Evaluation of the Risk of Recurrent Stroke

Each patient's risk of recurrent stroke was evaluated according to the Essen Stroke Risk Score (ESRS) (12–14). The ESRS was originally derived from cerebrovascular patients in the Clopidogrel vs. Aspirin in Patients at Risk of Ischemic Events (CAPRIE) trial as described previously (15, 16). ESRS is a 10-point scale: age 65–75 years (1 point), age > 75 years (2 points), arterial hypertension (1 point), diabetes mellitus (1 point), previous myocardial infarction (MI) (1 point), other cardiovascular disease (except MI and atrial fibrillation; 1 point), peripheral arterial disease (1 point), smoking (1 point), and previous transient ischemic attack (TIA) or ischemic stroke in addition to qualifying events (1 point).

### Evaluation of the Severity of Ischemic Stroke

The severity of AIS was assessed according to the National Institute of Health Stroke Scale (NIHSS). The NIHSS is widely used for this purpose, as described previously (17).

### Subtypes of Ischemic Stroke

The categorization of subtypes of ischemic stroke was mainly based on the Trial of ORG 10172 in Acute Stroke Treatment (TOAST) (18). The TOAST classification delineates five subtypes of ischemic infarction: large-artery atherosclerosis (LAA), cardiac embolism (CE), small-artery occlusion (SAO), stroke of other determined cause (SOC), and stroke of undetermined cause (SUC).

### Acquisition and Analysis of MRI Data

Within 72 h after admission, all participants underwent MRI scanning with 3.0-T scanners (Magnetom Avanto, HDxt, GE) using a dedicated head coil. Patients were placed in the supine position. The scanning protocol included DWI and its post-processing [apparent diffusion coefficient (ADC) sequence] with 5-mm-thick sections. The infarct volumes were calculated from DWI images. In DWI images, the infarct areas in each image were calculated separately and added to obtain the total infarct areas. Whole infarct volumes were obtained as infarct areas × thickness. Volume calculations on diffusion MRI were performed by a radiologist with 10 years of experience. The radiologist was blinded to the β2M and mRS data.

### Statistical Analyses

All statistical analyses were conducted with the Statistical Package for the Social Sciences version 19.0 (SPSS Co., Chicago, IL, USA). Continuous data were tested for normality using the Kolmogorov-Smirnov test. Several continuous variables that followed a normal distribution, such as Cr, uric acid (UA), total cholesterol (TC), and low-density lipoprotein (LDL), were expressed as the mean ± standard deviation. Other variables did not follow normal distributions and were presented as the median and the interquartile range (IQR). Categorical variables were expressed as constituent ratios. Differences in continuous variables between groups were assessed by ANOVA or the Mann-Whitney *U*-test. Differences in categorical variable distribution between groups were assessed by the χ^2^ test. Spearman correlation analysis was used to determine the correlation of β2M with NIHSS scores, infarct volumes, ESRS values, and mRS scores. The optimal cutoff value for the continuous β2M level was calculated by applying a receiver operating curve (ROC) analysis to test all possible cutoffs for discriminating between good and poor outcomes. Furthermore, we calculated the risk ratios (RRs) with 95% confidence intervals (CIs) for AIS 3-month outcome risk factors (the binary variables). Finally, we entered those possible risk factors into multiple regression models to avoid multicollinearity. All tests used a *P* value of 0.05 as a threshold for significance.

## Results

### Clinical and Demographic Data

A total of 205 patients with AIS were recruited in this study (four with missing data were excluded). None of the patients had recurrent stroke, and only two patients died within 3 months. As classified by mRS scores, 84 participants had good 3-month outcomes, and 121 participants had poor 3-month outcomes. Table 1 presents descriptive information on the study sample. The patients had a mean age of 70.76 ± 12.13 years (range, 22–94 years), and slightly more than half (58.0%) of the patients were male. Forty (19.5%) patients had a history of smoking, and 26 (12.7%) patients consumed alcohol. Most of the patients (68.8%) had hypertension. Small numbers of patients had type 2 diabetes (20.0%), a history of stroke (30.2%), and coronary heart disease (CHD) (13.7%). The level of β2M was 1.92 (0.66) mg/l. Overall, the median (IQR) NIHSS score and ESRS value were 6 (5) and 3 (1), respectively. The infarct volume was 1.71 (11.68) mm^3^. Compared to patients with good 3-month outcomes, those with poor outcomes had an increased prevalence of CHD; increased levels of Cr, urea, UA, cystatin c (CysC), and β2M; increased NIHSS scores; increased infarct volumes; and a reduced prevalence of smoking and alcohol use (all *P* < 0.05).

**Table 1:** Clinical and laboratory findings in patients with good and poor 3-month outcomes.

**Variable**	**mRS**		***P* value**
	**Good outcome (*N* = 84)**	**Poor outcome (*N* = 121)**	
Age (year)	69.29 ± 11.58	71.78 ± 12.45	0.149
Male, *n* (%)	54 (64.3)	65 (53.7)	0.132
Smoker, *n* (%)	22 (26.2)	18 (14.9)	0.044
Alcohol user, *n* (%)	16 (19.0)	10 (8.3)	0.023
Hypertension, *n* (%)	53 (63.1)	88 (72.7)	0.143
Type 2 diabetes, *n* (%)	15 (17.9)	26 (21.5)	0.523
Stroke history, *n* (%)	22 (26.2)	40 (33.1)	0.292
CHD, *n* (%)	4 (4.8)	24 (19.8)	0.002
SBP (mmHg)	147.06 ± 21.63	153.70 ± 26.27	0.057
DBP (mmHg)	85.96 ± 14.57	85.08 ± 16.24	0.691
Hcy (μmol/l)	11.75 (5.38)	12.40 (7.30)	0.106
FBG (mmol/l)	5.38 (1.51)	5.53 (2.19)	0.714
Cr (μmol/l)	70.31 ± 15.72	79.25 ± 19.45	<0.001
Urea (mmol/l)	5.41 ± 1.38	5.97 ± 1.86	0.015
UA (μmol/l)	315.12 ± 68.23	349.09 ± 88.33	0.002
LDL (mmol/l)	2.41 ± 0.68	2.41 ± 0.78	0.996
TG (mmol/l)	1.55 (1.03)	1.34 (1.18)	0.309
TC (mmol/l)	4.41 ± 0.78	4.32 ± 0.90	0.478
HDL (mmol/l)	1.43 ± 0.32	1.38 ± 0.34	0.253
VLDL (mmol/l)	0.31 (0.21)	0.28 (0.24)	0.513
CysC (mg/l)	1.06 ± 0.23	1.33 ± 0.32	<0.001
CRP (mg/l)	1.57 (3.21)	2.12 (6.57)	0.124
β2M (mg/l)	1.73 (0.38)	2.23 ± 0.562.16 (0.66)	<0.001
NIHSS score	4 (3)	8 (4)	<0.001
ESRS	2 (2)	3 (1)	0.234
Infarct volume (mm^3^)	1.32 (4.92)	3.47 (17.64)	0.003

*Quantitative data are expressed as the mean ± SD if normally distributed or as the median (interquartile range) if non-normally distributed. CHD, coronary heart disease; SBP, systolic blood pressure; DBP, diastolic blood pressure; Hcy, homocysteine; FBG, fasting blood glucose; Cr, serum creatinine; UA, uric acid; LDL, low-density lipoprotein cholesterol; TG, triglyceride; TC, total cholesterol; HDL, high-density lipoprotein cholesterol; VLDL, very low-density lipoprotein cholesterol; CysC, cystatin C; CRP, C-reactive protein; NIHSS, National Institute of Health Stroke Scale; ESRS, Essen Stroke Risk Score*.

### Association of Serum β2-Microglobulin With Essen Stroke Risk Score Values, National Institute of Health Stroke Scale Scores, Infarct Volumes, and Modified Rankin Scale Scores in Patients With Acute Ischemic Stroke

Table 2 shows the associations between β2M and ESRS values, NIHSS scores, infarct volumes, and mRS scores. The associations of β2M with the severity of AIS (NIHSS score), the 3-month outcome of AIS (mRS score), and the risk of recurrent cerebral infarction (ESRS) were analyzed using partial correlation analysis. The results showed that β2M levels were significantly and positively correlated with mRS scores (*r* = 0.402, *P* < 0.001) and ESRS values (*r* = 0.176, *P* < 0.001). However, β2M levels were not correlated with NIHSS scores (*r* = 0.080, *P* = 0.255) or infarct volumes (*r* = 0.013, *P* = 0.859). Nonetheless, infarct volumes and NIHSS scores were significantly and positively correlated with mRS scores.

**Table 2:** Correlation of β2-microglobulin (β2M) with infarct volume, National Institute of Health Stroke Scale (NIHSS) score, Essen Stroke Risk Score (ESRS), and modified Rankin Scale (mRS score) (*N* = 205).

	**β2M**	**Infarct volume**	**NIHSS score**	**ESRS**	**mRS score**
β2M	1	**–**	**–**	**–**	**–**
Infarct volume	0.013	1	**–**	**–**	**–**
NIHSS score	0.080	0.394^**^	1	**–**	**–**
ESRS	0.176^**^	0.035	0.085	**1**	**–**
mRS score	0.402^**^	0.169^*^	0.539^**^	0.087	**1**

* *P < 0.01*,

** *P < 0.001. The bold values mean that the correlation coefficients are statistically significant*.

### The Optimal Cutoff Value to Discriminate Between Good and Poor Outcomes at 3 Months After Acute Ischemic Stroke

**Figure 1:**
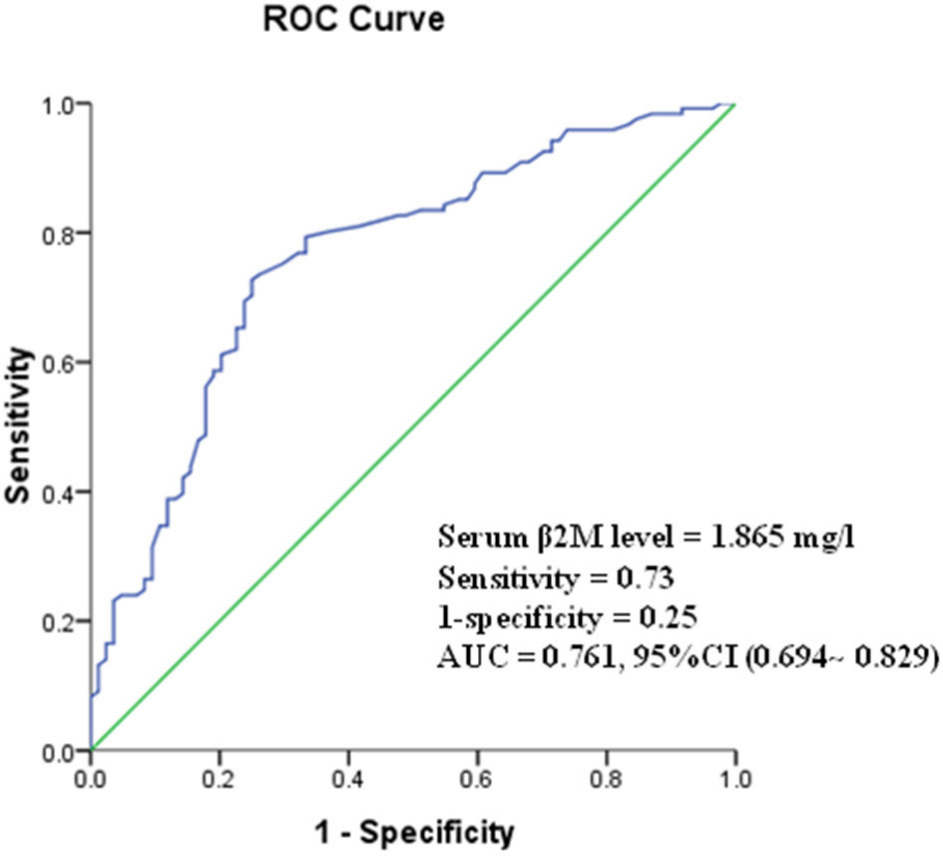
Receiver operating curve (ROC) showed predictive serum level of β2M for poor outcome.

A β2M value of 1.865 mg/l was calculated by ROC analysis as the optimal cutoff value to discriminate between good and poor outcomes in patients with AIS (area under the curve 0.761, 95% CI 0.694~0.829). The cutoff value had a sensitivity of 73% and a specificity of 75% for differentiating good outcomes from poor outcomes (Figure 1).

### β2-Microglobulin Was Associated With Poor Outcomes at 3 Months After Acute Ischemic Stroke

RR was calculated to evaluate the risk of poor outcome at 3 months for several binary variables (smoking, alcohol use, CHD, and dichotomized β2M). The value of β2M was changed to a binary variable using the cutoff value (1.865 mg/l). In Table 3, all the risk factors had significant cause-effect associations with poor outcome at 3 months after AIS.

**Table 3:** Risk factors for poor 3-month outcomes in acute ischemic stroke (AIS) patients (*N* = 205).

	**RR**	**95% CI**
Smoker	0.492	0.245~0.990
Alcohol user	0.383	0.164~0.892
CHD	4.948	1.649~14.853
β2M	8.000	4.237~15.105

We used multiple binary logistic regression models to analyze the association between risk factors and poor outcomes at 3 months after AIS. In model 1, β2M was adjusted for smoking, alcohol use, and CHD. In model 2, we added the variables of Cr, urea, UA, CysC, and infarct volume. Our results indicated that β2M was still significantly and positively associated with poor outcomes at 3 months after AIS in this study (*P* < 0.05) after adjusting for other risk factors (Table 4).

**Table 4:** Risk factors for poor 3-month outcomes in acute ischemic stroke (AIS) patients according to binary logistic regression analysis (*N* = 205).

	**Model 1**	**Model 2**
	**OR (95% CI)**	**OR (95% CI)**
Smoker	0.683(0.271–1.719)	0.580(0.207–1.622)
Alcohol user	0.612(0.206–1.822)	0.491(0.150–1.612)
CHD	2.564(0.780~8.434)	1.829(0.526–6.363)
Cr		1.003(0.978–1.029)
Urea		1.019(0.817–1.271)
UA		0.999(0.994–1.004)
CysC		12.255(1.797–83.574)
Infarct volume		1.017(1.001–1.033)
*β*2M	8.799(3.674–21.070)	3.325(1.089–10.148)

*Conditional binary logistic regression with backward selection*.

### β2-Microglobulin Was Associated With Trial of ORG 10172 in Acute Stroke Treatment Subtypes

Our data showed that the levels of β2M varied in different subgroups of AIS (TOAST classification) (*P* = 0.007). The Kruskal-Wallis test and *post hoc t*-tests were used to compare the level of β2M in each TOAST subtype. Pairwise comparison methods showed that there were significant differences between the CE and LAA groups (*P* = 0.002), the CE and SAO groups (*P* < 0.001), the CE and SOC groups (*P* = 0.04), and the CE and SUC groups (*P* = 0.023). However, the remaining subtypes were not significantly different (*P* > 0.05) (Table 5).

**Table 5:** Correlation analysis between β2-microglobulin (β2M) and Trial of ORG 10172 in Acute Stroke Treatment (TOAST) subtype (*N* = 205).

**Parameters**	**TOAST**					***P* value**
	**LAA**	**CE**	**SAO**	**SOC**	**SUC**	
*N*	90	26	75	9	5	
β2M (mg/l)	1.93 (0.68)	2.31 (0.77)	1.84 (0.60)	1.82 (0.55)	1.80 (1.10)	0.007

*The value of β2M is expressed as the median and IQR. The P value reflect a comparison between multiple groups (TOAST subgroups)*.

## Discussion

This cohort study investigated the relationships between serum β2M levels and NIHSS scores, mRS scores, and ESRS values in 205 patients with AIS. Our data revealed that serum β2M levels were significantly and positively correlated with ESRS values and mRS scores. Furthermore, we found that patients with high levels of serum β2M (>1.865 mg/l) were at high risk for poor outcomes at 3 months after AIS. Therefore, β2M may represent a prognostic predictor for the outcome and recurrence risk of AIS.

AIS is a type of ischemia/reperfusion injury caused by inadequate blood supply to the brain. In the damage period of AIS, the lack of oxygen and glucose supply can rapidly induce local neuron death through cytotoxicity, thus activating damage-associated molecular patterns (DAMPs). These inflammatory substances orchestrate focal inflammatory responses, catalyze tissue death, and worsen clinical outcomes (19). In our study, we found that patients with higher levels of serum β2M had a higher risk of poor outcomes at 3 months after AIS. This result suggests that β2M is associated with inflammation following AIS and thus determines the prognosis.

As a novel biological marker associated with inflammation, β2M has been studied extensively in autoimmune diseases, cancer, infection, kidney disease, peripheral artery disease, and cardiovascular disease (CVD) (20, 21). More importantly, β2M is a potential initiator that promotes the development of an inflammatory reaction (8). As atherosclerosis is a chronic inflammatory process (22), continuous inflammation promotes the development of atherosclerosis (23). Furthermore, lymphocytes and the surfaces of mononuclear cells are especially rich in β2M, which is synthesized in large amounts by lymphocytes and is regulated by interferons and pro-inflammatory monocytes (24). Given these prior results, we speculate that β2M plays a role in the pathophysiological development of atherosclerosis. The occurrence of AIS is one of the end point events of the pathological processes, including LAA, SAO, and CE, that define the TOAST subtypes (18). Interestingly, our data showed that levels of β2M varied among different subgroups of AIS (TOAST classification) (*p* = 0.007), indicating that the relationship between β2M and subtypes of AIS was very complicated in terms of etiology and was not to arteriosclerosis. For example, serum β2M was significantly higher in the CE group than in other groups in our cohort, which suggests that CE is the strongest cause of elevated β2M levels in patients with AIS. The literature shows that CE is the most common subtype of AIS (25), and the most common cause of CE is atrial fibrillation (AF), with the remaining subtypes being myocardial infarction and cardiomyopathy, which are closely associated with chronic inflammation (26). A recent report has shown that inflammation is directly related to AF and that AF is a direct result of inflammation (27). In particular, the CE subtype showed significantly higher median plasma levels of inflammatory cytokines than other types of AIS (28). In addition, Zeng et al.'s study also showed that the pathogenesis of LAA was more strongly activated by inflammation than that of SAO, which was associated with the changes in CRP (29). These studies are consistent with our findings and confirmed the inflammatory characteristics of β2M. However, the specific characteristics of the relationship between β2M and each subtype of AIS merit further study.

The results of our study showed that serum β2M levels were significantly and positively correlated with ESRS. Many criteria used to calculate the ESRS, such as myocardial infarction, cardiovascular diseases, peripheral arterial disease, TIA, and cerebral infarction, are among the etiological bases of AIS (30–32). All of the above studies indicate that β2M may be closely related to the etiology of AIS. As a new marker of chronic inflammation *in vivo*, β2M is a significant and valuable predictor of the survival of some populations and the prognosis of some diseases (33, 34). In particular, the results of a recent prospective study showed that β2M was closely related to the risk of AIS among women (10). However, the fact that ESRS is associated does not directly support the role of ESRS in predicting ischemic stroke, and this link should be confirmed in further studies with longer follow-up for stroke recurrence. However, as a biomarker, β2M may provide some meaningful information for patients at high risk for recurrent AIS.

As a biomarker closely related to immunity and inflammation, β2M concentrations were found to be significantly elevated in the cerebrospinal fluid of patients with multiple sclerosis (MS) (35). This result suggests that β2M is closely related to central nervous system inflammation. Recently, the impact of MHC I of the development of the nervous system has attracted researchers' attention, as MHC I plays an important role in regulating axon growth and cortical connections (36). Furthermore, TNF-α and γ-interferon can promote the expression of MHC I and β2M in the central nervous system (37). AIS is characterized by the rapid activation of microglia and time-dependent peripheral immune cell activation and infiltration into damaged brain tissue; then, ischemic brain injury occurs, caused by local inflammation (38, 39). At the same time, inflammation also has positive aspects, such as the regeneration of neurons and the alleviation of ischemic damage (for instance, the phenotype of microglia shifts from M1 to M2), especially in the subacute and recovery periods of AIS (40, 41). MHC I in the central nervous system is also involved in the process of AIS in the context of inflammation (42). As a light chain of MHC I, β2M is co-expressed with it (43), and β2M is an important component for the stable presence of MHC I protein on the cell surface (44). These studies indicate that β2M may play an important role in the inflammatory process of brain injury and recovery during the AIS period. At the same time, these studies provide a meaningful explanation for our findings regarding the association between β2M and outcomes of AIS. In our research, we found a puzzling phenomenon: serum β2M levels were associated with mRS scores, but they were not associated with the NIHSS scores or infarct volumes of AIS patients at admission, which seems paradoxical. In a literature search, we found that the most common post-stroke infections are stroke-associated pneumonia (SAP) and urinary tract infections (UTIs), both of which are closely associated with the outcome of AIS (45). According to the study by Shim et al., the brain attempts to reduce damage through the cerebral immune defense mechanism (immunosuppression) after stroke, but this occurs at the expense of an increased susceptibility to infection (stroke-associated infection) (46). NIHSS scores and infarct volume are closely associated with outcomes in patients with AIS. However, the values of these parameters in our study were recorded only at admission and may change dynamically with the progression of AIS. For example, some patients in our study may develop the complication of stroke-associated infection during the acute period of AIS; this complication has a direct and serious influence on the prognosis of AIS (45). Due to the influence of this factor, the values of these indicators (NIHSS score and infarct volume) at admission may not fully reflect patients' prognosis. These may be the explanations for the paradoxical phenomenon in our study. As one of the main components of MHC I, β2M is closely related to immunity and inflammation (4, 8) and plays a key role in post-stroke infection (5, 47, 48). However, little has been reported on the relationship between β2M and stroke-associated infection, especially the role of β2M in the immune regulation of stroke. In our next study, we will stratify the patients (the patients will be divided into two groups according to the presence or absence of stroke-associated infection) and further investigate the relationship between β2M and post-stroke infection as well as the prognosis of AIS.

Intravenous thrombolysis with alteplase is currently considered an effective method for the treatment of AIS (3). However, due to the limited time window (<4.5 h), this therapy benefits only a small number of patients. Additionally, intravenous alteplase has some potential complications, such as hemorrhage transformation, which can result in a poor prognosis. As a new approach to the treatment of AIS, immunotherapy has attracted great scientific attention. Blocking specific inflammatory pathways after stroke may delay brain tissue damage (49) and extend time windows for treatment. Studies have shown that some proteins can activate human β2M, leading to the inhibition of MHC I expression, and can ultimately downregulate class I-mediated antigen presentation. For example, ESAT-6 is an abundantly secreted protein of *Mycobacterium tuberculosis* (50). As previously reported, inhibiting the expression of MHC I molecules is a method of protecting the brain after AIS (42). Therefore, further studies of β*2M* may provide some useful clues for effective treatment strategies for AIS.

## Limitations

First, the relatively low number of research subjects is an important limitation of this study. Second, data from multiple medical centers are needed to confirm our results. Third, the biochemical indicators were not remeasured 3 months after AIS. Finally, our follow-up spanned only 3 months, whereas 1 year or longer would have been ideal. In the future, we will increase the sample size and follow-up time and conduct a multicenter case-control study to overcome the limitations of the current study.

## Conclusion

In conclusion, serum β2M is a potential biomarker for the recurrence risk and 3-month outcome of AIS, but it is not directly related to the severity of AIS or the size of the infarct at admission.

## Data Availability Statement

The datasets generated for this study are available on request to the corresponding author.

## Author Contributions

SQ was involved in the design of the study, data collection, interpretation of the data, manuscript writing, and was a recipient of the obtained funding. FH took part in the design of the study, data collection, the statistical analysis, and was a recipient of the obtained funding. QT was a recipient of the obtained funding and was involved in the interpretation of the data and the manuscript revision. JWu, JZ, ZC, XW, QL, and JWa were involved in the data collection. WG participated in the data analysis, interpretation of the data, and the manuscript revision.

### Conflict of Interest

The authors declare that the research was conducted in the absence of any commercial or financial relationships that could be construed as a potential conflict of interest.
